# A grading system that predicts the risk of dialysis induction in IgA nephropathy patients based on the combination of the clinical and histological severity

**DOI:** 10.1007/s10157-018-1657-0

**Published:** 2018-10-26

**Authors:** Hideo Okonogi, Tetsuya Kawamura, Kensuke Joh, Kentaro Koike, Yoichi Miyazaki, Makoto Ogura, Nobuo Tsuboi, Keita Hirano, Masato Matsushima, Takashi Yokoo, Satoshi Horikoshi, Yusuke Suzuki, Takashi Yasuda, Sayuri Shirai, Takanori Shibata, Motoshi Hattori, Yuko Akioka, Ritsuko Katafuchi, Akinori Hashiguchi, Satoshi Hisano, Akira Shimizu, Kenjiro Kimura, Shoichi Maruyama, Seiichi Matsuo, Yasuhiko Tomino, for The Special IgA Nephropathy Study Group

**Affiliations:** 1 Division of Nephrology and Hypertension, Department of Internal Medicine, Jikei University School of Medicine, 3-25-8, Nishi-Shimbashi, Minato-ku, Tokyo, 105-8461 Japan; 2 Department of Pathology, Tohoku University Graduate School of Medicine, Sendai, Japan; 3 Division of Clinical Epidemiology, General Research Center of Medicine, Jikei University School of Medicine, Tokyo, Japan; 4 Division of Nephrology, Department of Internal Medicine, Faculty of Medicine, Juntendo University, Tokyo, Japan; 5 Department of Internal Medicine, Kichijoji Asahi Hospital, Tokyo, Japan; 6 Division of Kidney and Hypertension, Department of Internal Medicine, St. Marianna University School of Medicine, Kawasaki, Japan; 7 Division of Nephrology, Department of Internal Medicine, Showa University School of Medicine, Tokyo, Japan; 8 Department of Pediatric Nephrology, Tokyo Women’s Medical University School of Medicine, Tokyo, Japan; 9 Kidney Unit, National Fukuoka-Higashi Medical Center, Fukuoka, Japan; 10 Department of Pathology, Keio University School of Medicine, Tokyo, Japan; 11 Department of Pathology, Faculty of Medicine, Fukuoka University, Fukuoka, Japan; 12 Department of Analytic Human Pathology, Nippon Medical School, Tokyo, Japan; 13 Division of Nephrology, Department of Internal Medicine, Faculty of Medicine, University of Nagoya, Nagoya, Japan

**Keywords:** Histological classification, Clinical classification, Renal biopsy, IgA nephropathy, Receiver-operating characteristic analysis

## Abstract

Histological classification is essential in the clinical management of immunoglobulin A nephropathy (IgAN). However, there are limitations in predicting the prognosis of IgAN based on histological information alone, which suggests the need for better prognostic models. Therefore, we defined a prognostic model by combining the grade of clinical severity with the histological grading system by the following processes. We included 270 patients and explored the clinical variables associated with progression to end-stage renal disease (ESRD). Then, we created a predictive clinical grading system and defined the risk grades for dialysis induction by a combination of the clinical grade (CG) and the histological grade (HG). A logistic regression analysis revealed that the 24-h urinary protein excretion (UPE) and the estimated glomerular filtration rate (eGFR) were significant independent variables. We selected UPE of 0.5 g/day and eGFR of 60 ml/min/1.73 m^2^ as the threshold values for the classification of CG. The risk of progression to ESRD of patients with CG II and III was significantly higher than that of patients with CG I. The patients were then re-classified into nine compartments based on the combination of CG and HG. Furthermore, the nine compartments were grouped into four risk groups. The risk of ESRD in the moderate, high, and super-high-risk groups was significantly higher than that in the low-risk group. Herein, we are giving a detailed description of our grading system for IgA nephropathy that predicted the risk of dialysis based on the combination of CG and HG.

## Introduction

Immunoglobulin A nephropathy (IgAN) is the most prevalent form of primary chronic glomerulonephritis; 20–40% of IgAN patients progress to end-stage renal disease (ESRD) within 20 years from its onset [1, 2]. Numerous studies have identified histological and clinical prognostic parameters. Most previous studies have reported that the histological grade, severe proteinuria, and a reduced renal function were strong predictors of progression. Some studies have suggested that hypertension, age, and gender were also prognostic factors. Histological classification for evaluating the disease severity and deciding therapeutic strategies is essential in the clinical management of IgAN [3–5]. Recently, an international working group created the Oxford classification of IgAN [6]. The Oxford classification, which was consensus-based, defined pathologic lesions with acceptable interobserver reproducibility and identified four prognostic pathologic features based on a rigorous statistical analysis (mesangial hypercellularity, endocapillary hypercellularity, segmental glomerulosclerosis, and tubular atrophy/interstitial fibrosis). However, the results of the validation studies remain controversial [5], and most recently, a multicenter study proposed addition of crescents scores to the original Oxford/MEST classification [7].

In our country, IgAN is often diagnosed in patients with a relatively early stage of the disease who show asymptomatic proteinuria with microhematuria or isolated microhematuria. In *the Clinical Practice Guidelines for IgA Nephropathy*, treatments with renin–angiotensin system (RAS) inhibitors or corticosteroids were recommended to patients with sustained proteinuria of > 0.5 g/day [8, 9]. Severe proteinuria (≥ 1 g/day) at the time of renal biopsy (RBx) is a well-known prognostic factor of IgAN [10, 11]. On the other hand, in the Oxford cohort, patients had severe proteinuria (mean, 1.7 g/day) and the majority of patients were white race [6]. The Special IgAN Study Group of the Progressive Renal Diseases (IgAN-SG) developed an evidence-based histological classification of IgAN that was suitable for predicting long-term renal outcome of IgAN in Japan, because the optimal threshold values and classifications could differ according to the patient background and the outcome definitions in different cohorts [12]. This Japanese histological classification system demonstrated that pathological lesions that independently predicted the progression to ESRD were global sclerosis, segmental sclerosis, and fibrous crescents in IgAN patients who required dialysis within < 5 years after biopsy (early progressors) and cellular/fibrocellular crescents for those who required dialysis at 5–10 years after biopsy (late progressors). The classification included four histological grades, which identified the magnitude of the risk of progression to ESRD. This classification was validated by Sato et al. and was well-correlated with the long-term prognosis in their cohorts [13]. In our previous study, however, 11 patients with a histological grade (HG) I, which indicates the lowest risk of progression to ESRD (percentage of glomeruli exhibiting cellular/fibrocellular crescents, global sclerosis, segmental sclerosis or fibrous crescents vs. total glomeruli < 25%) developed ESRD over the long-term follow-up period [12]. The fact that these HG I progressed to ESRD indicated that there are limitations in predicting the prognosis of IgAN based on the histological variables at the initial diagnosis alone, and suggested the need to create better prognostic models.

To create better prognostic models, the special IgAN-SG at first established a predictive grading system for assessing the clinical severity based on the clinical variables associated with progression to ESRD. Furthermore, we defined a prognostic model for predicting the risk of dialysis induction by combining the grade of clinical severity with the histological grading system, and reported the prognostic model in clinical guides for IgAN [9]. The essence of the model was referred by *the Clinical Practice Guidelines for IgA Nephropathy* [8]. However, details of the process of constructing the grading system have not been published yet. Therefore, in this special report, we described the process of constructing these grading systems, including the details of the statistical analyses.

## Process of constructing the grading system

### Patient selection and measurements

The protocol of this analysis was the same as our previous study [12]. Briefly, the multicenter retrospective case–control study was conducted in collaboration with 16 hospitals. Primary IgAN was diagnosed based on the detection of IgA-dominant mesangial immune deposits. Patients with systemic diseases were excluded. The inclusion criteria were as follows: (1) the detection of > 10 glomeruli in a paraffin section under light microscopy; (2) the patient progressed to ESRD requiring chronic dialysis or was followed for at least 5 years after renal biopsy without the need for dialysis; and (3) the patient’s clinical course and therapies including—but not limited to—corticosteroids, renin–angiotensin system (RAS) inhibitors, immunosuppressive drugs, and tonsillectomy, before and after renal biopsy were available. Primary IgAN patients satisfying these criteria were registered from each hospital. Renal biopsies were performed from February 1980 to January 2002.

The clinical and laboratory characteristics of the patients are shown in Table 1. Among 287 patients in our previous study [12], 270 patients who had no missing values for 24-h urinary protein excretion (UPE) or eGFR at the time of RBx were analyzed to construct this grading system. At the end of the follow-up period, the UPE data, whose mean value was 0.92 g/day, were available from 209 patients. Forty-eight patients (18%) progressed to ESRD during the follow-up period. In addition, the patients were stratified according to their prognosis. A number of patients, age, UPE, serum creatinine, eGFR, serum uric acid, number of patients with hypertension and mean arterial pressure at the time of RBx, and observation period at the end of follow-up were significantly different between ESRD (+) group and ESRD (−) group.

**Table 1:** Clinical and laboratory characteristics of the patients

Characteristic	Whole patients	Prognosis		
		ESRD (+)	ESRD (−)	*p*
Number of patients [Male (%)]	270 [136 (50)]	48 [32 (67)]	222 [104 (47)]	0.017
At the time of biopsy				
Age (years)	35.6 ± 14.1	41.2 ± 15.2	34.4 ± 13.6	0.002
Number of patients < 18 years of age (%)	26 (9.6)	4 (8.3)	22 (9.9)	0.798
Duration from onset to biopsy (months) (IQR)	30 (12–72)	48 (12–86)	24 (11–72)	0.312
UPE (g/day) (IQR)	0.81 (0.37–1.80)	1.80 (1.07–3.31)	0.70 (0.30–1.50)	< 0.001
SCr (mg/dl)	0.93 ± 0.63	1.54 ± 1.18	0.80 ± 0.29	< 0.001
eGFR (ml/min/1.73 m^2^)	79.8 ± 30.6	55.8 ± 28.5	85.0 ± 28.5	< 0.001
SUA (mg/dl)	5.94 ± 1.49	6.67 ± 1.44	5.79 ± 1.46	0.001
Number of patients with hypertension (%)	89 (33)	26 (54)	63 (28)	0.001
MAP (mmHg)	93.9 ± 15.7	103.3 ± 16.2	91.9 ± 14.9	< 0.001
Number of patients with severe hematuria (%)	72 (31)	6 (18)	66 (34)	0.072
At the end of follow-up				
Observation period (years) (IQR)	10.0 (6.3–12.6)	7.6 (4.2–11.4)	9.7 (6.7–12.8)	< 0.001
Number of patients with ESRD (%)	48 (18)	48 (100)	0 (0)	

The values are expressed as the number (%), median (IQR), or mean ± SD. Hypertension was defined as a systolic blood pressure of ≥ 140 mmHg or a diastolic blood pressure of ≥ 90 mmHg, and/or taking antihypertensive drugs at RBx. Severe microscopic hematuria was defined based on the presence of ≥ 100 urinary erythrocytes per high-power field [12]. The patients were stratified according to their prognosis. Difference of baseline characteristics between ESRD (+) group and ESRD (−) group was examined using *t* test, Mann–Whitney *U* test, or Chi-square test

*eGFR* estimated glomerular filtration rate, *ESRD* end-stage renal disease, *IQR* interquartile range, *MAP* mean arterial pressure, *SCr* serum creatinine, *SUA* serum uric acid, *UPE* 24-h urinary protein excretion

### The construction of a system for grading the risk of dialysis induction using the combination of histological and clinical severity

**Fig. 1:**
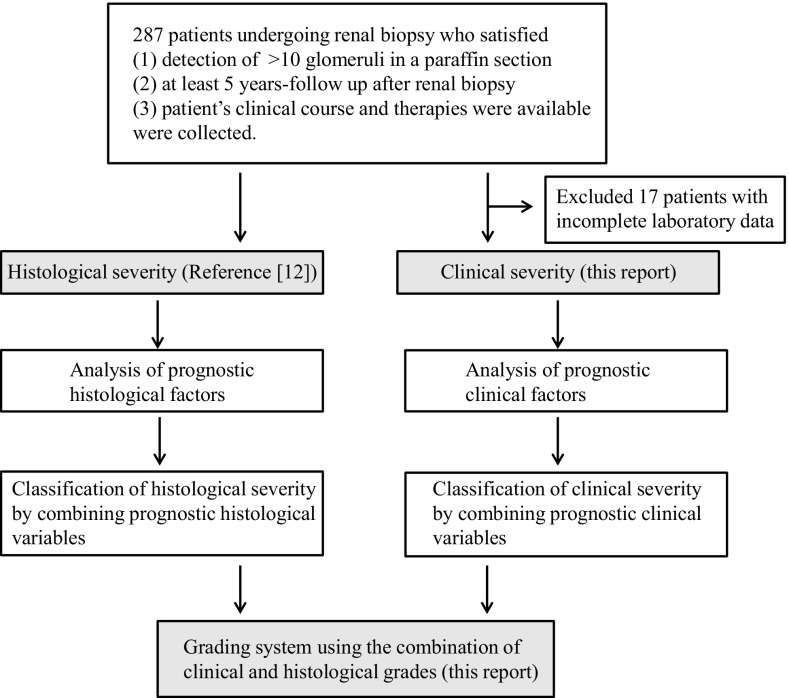
Process of constructing the grading system that predicts the risk of dialysis induction in IgA nephropathy patients. The inclusion criteria, steps of constructing histological classification, clinical classification, and grading system are shown

A system for grading the risk of dialysis induction of IgAN was constructed as follows, and Fig. 1 also shows the process by flowchart.

### Step 1: The histological classification of IgAN predicting the risk of dialysis induction

First, the special IgAN-SG at first established the histological classification of IgAN [12]. Briefly, the association between pathological variables and the incidence of subsequent ESRD were examined using multivariate logistic regression analysis separately in patients who required dialysis earlier than 5 years (Early Progressors) and those who required dialysis within 5 to 10 years (Late Progressors) after RBx. Independent pathological variables predicting to ESRD were global sclerosis, segmental sclerosis and fibrous crescents for Early Progressors, and global sclerosis and cellular/fibrocellular crescents for Late Progressors. Four histological grades, HG I, HG II, H III, and HG IV, were established corresponding to < 25%, 25–49%, 50–74%, and 75% ≤ of glomeruli exhibiting cellular of fibrocellular crescents, global sclerosis, segmental sclerosis, or fibrous crescents. Eleven (7%) patients in HG I, 12 (16%) in HG II, 13 (31%) in HG III and 13 (68%) in HG IV progressed to ESRD. Multivariate logistic analysis revealed that the risk of progression to ESRD was significantly higher in HG II, III, and IV than in HG I [odds ratio (OR) (95% confidence interval), 2.4 (1.02–5.79), 5.7 (2.33–13.99) and 27.6 (8.77–86.69) vs. 1.0].

### Step 2: The analysis of factors associated with renal progression

Second, to evaluate clinical factors associated with progression to ESRD, we examined the association between the clinical variables at the time of RBx with the incidence of subsequent ESRD using univariate and multivariate logistic regression analyses. As shown in Table 2, the univariate logistic regression analysis revealed that UPE, eGFR, SUA, age, gender (male), and hypertension were significantly associated with progression to ESRD, while the multivariate logistic regression analysis showed that UPE and eGFR were independently associated with progression to ESRD (Model 1). To elucidate the effects of individual treatments on the predictive values of selected variables, we adjusted these variables for the use of both corticosteroids and RAS inhibitors (Model 2) during the follow-up period. As a result, UPE and eGFR at the time of RBx were still independently associated with progression to ESRD.

**Table 2:** Association between the clinical parameters and progression to ESRD

Variables	Univariate analysis			Multivariate analysis					
	OR	95% CI	*p*	Model 1			Model 2		
				OR	95% CI	*p*	OR	95% CI	*p*
UPE, per 1 g/day	1.70	1.38–2.09	< 0.001	1.69	1.29–2.20	< 0.001	1.61	1.20–2.17	0.002
eGFR, per 10 ml/min/1.73 m^2^	0.65	0.56–0.76	< 0.001	0.64	0.50–0.83	0.001	0.64	0.49–0.84	0.001
SUA, per 1 mg/dl	1.50	1.17–1.92	0.001	1.19	0.84–1.69	0.323	1.20	0.84–1.71	0.316
Age, per 10 years	1.42	1.13–1.79	0.003	0.66	0.43–1.02	0.059	0.69	0.44–1.08	0.103
Gender (male)	2.27	1.18–4.37	0.014	0.88	0.31–2.50	0.803	0.91	0.31–2.62	0.855
Hypertension (yes)	3.11	1.63–5.92	0.001	1.20	0.43–3.35	0.728	1.28	0.45–3.66	0.646
Severe hematuria (yes)	0.422	0.17–1.07	0.069	0.43	0.14–1.40	0.162	0.40	0.12–1.31	0.130
Corticosteroids (yes)	2.57	1.36–4.86	0.004				1.52	0.53–4.35	0.438
RAS inhibitors (yes)	1.92	0.85–4.33	0.117				0.65	0.15–2.89	0.572

The univariate and multivariate analyses of the factors associated with progression to ESRD were performed using logistic regression

*CI* confidence interval, *eGFR* estimated glomerular filtration rate, *ESRD* end-stage renal disease, *OR* odds ratio, *RAS* renin–angiotensin system, *SUA* serum uric acid, *UPE* 24-h urinary protein excretion

### Step 3: The classification of clinical severity by combining UPE and the eGFR

Third, to assess the accuracy of the selected clinical prognostic variables, we performed a receiver-operating characteristic (ROC) analysis. As a result, UPE and eGFR had an area under the ROC curve value of 0.774 and 0.777, respectively. We then selected the typical threshold values for UPE and eGFR, and the sensitivity and specificity were calculated for the individual threshold values (Table 3). We further calculated the positive likelihood and negative likelihood (Table 3). Next, we applied the candidate UPE values and eGFRs to a prognostic-predictive equation [threshold score (− 1.86) = 0.722 + 0.364 × UPE − 0.046 × eGFR] which was associated with the risk of future ESRD in 116 Japanese IgAN patients in a previous report [14]. In this formula, –1.86 was the threshold score that suggested better prognostic-predictive accuracy in the analysis [14]. The combination of UPE 0.5 g/day and eGFR 60 ml/min/1.73 m^2^ was one of the combinations that provided a suitable prognostic-predictive equation. We, therefore, selected UPE 0.5 g/day and eGFR 60 ml/min/1.73 m^2^ as the threshold values for the following analyses.

**Table 3:** ROC analysis of the risk of progression to ESRD

A. UPE				
UPE (g/day)	Sensitivity (%)	Specificity (%)	Positive likelihood ratio	Negative likelihood ratio
0.5	95.8	39.5	1.58	0.11
1.0	81.3	62.7	2.18	0.30
2.0	44.8	82.2	2.52	0.67

B. eGFR				
eGFR (ml/min/1.73 m^2^)	Sensitivity (%)	Specificity (%)	Positive likelihood ratio	Negative likelihood ratio
90	87.5	37.6	1.40	0.33
60	61.4	83.3	3.68	0.46
30	27.0	97.1	9.31	0.75

The UPE and eGFR had an area under the ROC curve of 0.774 and 0.777, respectively. The sensitivity and specificity values were obtained by an ROC analysis. The positive likelihood ratio was calculated by the following equation: Sensitivity/(1-Specificity). The negative likelihood ratio was calculated by the following equation: (1-Sensitivity)/Specificity

*eGFR* estimated glomerular filtration rate, *ESRD* end-stage renal disease, *RAS* renin–angiotensin system, *ROC* receiver-operating characteristic, *SUA* serum uric acid, *UPE* 24-h urinary protein excretion

Fourth, the patients were classified according to the combination of threshold values of the prognostic variables. The patients were classified into four classes according to the combination of UPE 0.5 g/day and eGFR 60 ml/min/1.73 m^2^ (Table 4). There was a significant difference in the incidence of ESRD among the four classes (*p* < 0.001). Moreover, the incidence of ESRD in Class 4 was significantly higher than that in Classes 1 (*p* < 0.001) and 3 (*p* < 0.001). Because none of the patients in Class 2 progressed to ESRD, we combined Class 1 and Class 2. Thus, the patients were re-classified into three clinical grades (CGs) (CG I, CG II, and CG III) (Table 4). The patients in CG I were assigned as the reference group in this analysis. The patients in CG II and CG III were found to have significantly higher ORs (6.4, *p* < 0.01; and 42.5, *p* < 0.001, respectively), in comparison with the reference group (Table 4).

**Table 4:** Classification of patients according to the clinical parameters

Class	UPE (g/day)	eGFR (ml/min/1.73 m^2^)	Incidence of ESRD (%)	Clinical grade	OR (95% CI)	*p*
Class-1	< 0.5	60 ≤	2/80 (2.5)	C-Grade I	Reference	
Class-2	< 0.5	< 60	0/7 (0)			
Class-3	0.5≤	60 ≤	16/123 (13)	C-Grade II	6.4 (1.4–28.4)	0.015
Class-4	0.5≤	< 60	30/60 (50)^a,b^	C-Grade III	42.5 (9.6–189)	< 0.001

The patients were classified into four classes according to the combination of the UPE level and the eGFR at the time of RBx. The incidence of ESRD is shown as the number of patients who progressed to ESRD/number of patients. The difference in the incidence of ESRD among the four classes was examined using the Kruskal–Wallis test (*p* < 0.001). Inter-group comparisons in classes were examined using the Steel–Dwass test; statistical significance was indicated as follows: ^a^*p* < 0.001, Class-1 vs. Class 4; ^b^*p* < 0.001, Class-3 vs. Class-4

The patients were re-classified into three clinical grades (GGs) by combination of the UPE level and the eGFR at the time of RBx. Class-1 + Class-2, Class-3, and Class-4 were equivalent to CG I, CG II, and CG III, respectively. The OR was determined by a logistic regression analysis

*CI* confidence interval, *eGFR* estimated glomerular filtration rate, *ESRD* end-stage renal disease, *OR* odds ratio, *RBx* renal biopsy, *UPE* 24-h urinary protein excretion

### Step 4: A grading system using the combination of clinical and histological grades to predict the risk of dialysis induction

Finally, we constructed a system for grading the risk of dialysis induction based on the combination of the grade of clinical severity and the HG. To construct a grading system, we modified four HGs (HG I, HG II, HG III, and HG IV) in the original study [12] into three HGs by combining HG III and HG IV, because the number of patients in HG IV was considerably low (6.6%), and the patients in both HG III and HG IV had a higher risk of progression to ESRD. Thus, the patients were classified into nine compartments by combining three CGs (CG I, CG II, and CG III) with three HGs (HG I, HG II, and HG III + IV) (Table 5). The incidence of ESRD in “HG I and CG I” was 1.4%; this compartment was assigned as the reference group in the subsequent analyses. The ORs of each compartment in comparison with the reference group were then calculated by logistic regression analyses. Consequently, as CG (HG) advanced in patients with the same HG (CG), the ORs were observed to increase incrementally in both directions. The “HG III + IV and CG III” compartment showed the highest risk of ESRD; the patients in this compartment had an OR of 130 (*p* < 0.001).

**Table 5:** Classification and the risk of progression to ESRD according to the combination of the clinical grade and the histological grade

Clinical grade	Histological grade		
	H-Grade I	H-Grade II	H-Grade III + IV
C-Grade I			
Incidence of ESRD (%)	1/72 (1.4)	0/10 (0)	1/5 (20)
OR (95% CI)	Reference	0	17.8 (0.9–339)
Risk groups	Low risk (Reference)	Moderate risk	High risk
C-Grade II			
Incidence of ESRD (%)	7/64 (11)	6/41 (15)	3/18 (17)
OR (95% CI)	8.7 (1.0–73)	12.2 (1.4–105)	14.2 (1.4–146)
Risk groups	Moderate risk	Moderate risk	High risk
C-Grade III			
Incidence of ESRD (%)	2/5 (40)	6/21 (29)	22/34 (65)
OR (95% CI)	47.3 (3.3–679)	28.4 (3.2–254)	130 (16-1058)
Risk groups	High risk	High risk	Super-high risk

The patients were classified into nine compartments according to the combination of the clinical grade and the histological grade. Then, nine compartments were grouped into four groups with similar ORs. The OR was examined by a logistic regression. The incidence of ESRD is shown as the number of patients who progressed to ESRD/number of patients. The OR was determined by a logistic regression analysis. The table was re-described from reference [8, 9] and modified

*CI* confidence interval, *ESRD* end-stage renal disease, *OR* odds ratio

Furthermore, we grouped the 9 compartments into 4 risk groups with similar ORs [i.e., low risk (reference), moderate risk (OR < 14), high risk (14 ≤ OR < 50), and super-high risk (50 ≤ OR)] (Table 5). In the present study, none of the patients in “HG II and CG I” progressed to ESRD. However, we assigned this compartment as a moderate risk group, because the ORs gradually increased as HG advanced in patients with CG II or CG III. The incidence of ESRD in each risk group and the OR of each risk group in comparison with the reference group (low-risk group) are calculated and are summarized in Table 6. Thus, the patients in the moderate, high, and super-high-risk groups had ORs of 9.0 (*p* < 0.05), 23.0 (*p* < 0.01), and 130 (*p* < 0.001), respectively.

**Table 6:** OR for the risk of the progression to ESRD in the four risk groups

Risk group	Number of patients	Incidence of ESRD (%)	OR (95% CI)	*p*
Low risk	72	1 (1.4)	Reference	
Moderate risk	115	13 (11)	9.0 (1.16–70.7)	0.036
High risk	49	12 (24)	23.0 (2.89–184)	0.003
Super-high risk	34	22 (65)	130 (16.0–1058)	< 0.001

Incidence of ESRD and ORs in each risk group is shown. The OR progressively increased from the low-risk group to the super-high-risk group. The OR was determined by a logistic regression analysis

*CI* confidence interval, *ESRD* end-stage renal disease, *OR* odds ratio

The clinical and histological characteristics, and the percentage of the patients treated with corticosteroids, RAS inhibitors, or immunosuppressive drugs during the follow-up period are summarized in Table 7. From the low-risk group to the super-high-risk group, severity of both clinical and histological characteristics progressively increased. Likewise, the percentage of patients receiving corticosteroids gradually increased as the risk of dialysis induction increased, with 67% of the patients in the super-high-risk group receiving corticosteroids.

**Table 7:** Summary of the clinical and histological characteristics and the treatments during the follow-up period

	Clinical characteristics		Histological characteristics	Treatments (%)			
Risk group	UPE (g/day)	eGFR (ml/min/1.73 m^2^)	Number of patients of H-grade (H-Grade I, II, III + IV)	Corticosteroids	RAS inhibitors	Immunosuppressive drugs	Tonsillectomy
Low risk	0.25 ± 0.13	98.5 ± 25.9	72, 0, 0	13	49	1.4	4.2
Moderate risk	1.52 ± 1.38	87.6 ± 24.9	64, 51, 0	40	78	2.6	7.8
High risk	1.66 ± 1.17	61.6 ± 23.2	5, 21, 23	47	90	0	6.1
Super-high risk	2.78 ± 1.89	40.4 ± 14.0	0, 0, 34	67	91	12	0

The clinical and histological characteristics and the percentage of the patients who received the treatment during the follow-up period in each risk group are shown. The values of UPE and eGFR are expressed as mean ± SD

*eGFR* estimated glomerular filtration rate, *UPE* 24-h urinary protein excretion, *RAS* renin–angiotensin system

## Discussion and comments

The present special report provided the process of constructing the grading system for the risk of dialysis by combining the grade of clinical severity with the histological grading system [12]. To the best of our knowledge, no grading system for IgAN has included both the clinical and histological grades (Table 5).

So far, several representative histological grading systems have been reported; however, there are some differences in included histological lesions among these reports (Table 8). Haas et al. [15], Manno et al. [16], and the Oxford classification in 2009 [6] evaluated glomerular and tubulo-interstitial lesions. Likewise, recently reported the Oxford classification in 2016 [7, 17] also evaluated glomeruar and tubulo-interstitial lesions. Katafuchi et. al. examined glomerular, tubulo-interstitial, and vascular lesions; then, they concluded that glomerular score more closely related to renal outcome than those according to total score including glomerular, tubulo-interstitial, and vascular lesions [18]. On the other hand, Lee et al. constructed the refined HS Lee grading system focusing simply on glomerular lesions [19], like as our histological classification system [12]. As the reasons for constructing histological grading system using only glomerular lesions, Kawamura et al. discussed that global sclerosis had a high statistically significant association with interstitial fibrosis, and global sclerosis showed outstanding reproducibility in the Oxford classification [12].

**Table 8:** Comparison of included histological lesions among histological grading systems

Reference	Histological lesions						
	Glomerular lesions					Tubulo-interstitial lesions	Vascular lesions
	Mesangial cellularity	Endocapillary proliferation	Glomerulosclerosis	Focal segmental sclerosis	Crescent		
Haas et al. [15]	×	×	×	×	×	×	
Manno et al. [16]	×	×	×	×	×	×	
Oxford classification 2009 [6]	×	×		×		×	
Oxford classification 2016 [7, 17]	×	×		×	×	×	
Katafuchi et al. [18]	×		×	×	×	×	×
Lee et al. [19]	×		×	×	×		
Kawamura et al. [12]			×	×	×		

*×* included histological lesion

Interestingly, when the patients in HG I, which showed the best prognosis among the four HGs, were classified into three classes according to the three CGs, the ORs for the risk of progression to ESRD increased as the CG advanced (ORs of [HG I − CG I], [HG I − CG II] and [HG I − CG III] were 1 (Reference), 8.7 and 47.3, respectively.). Likewise, when the patients in HG III + HG IV, whose histological classification was associated with a poor prognosis [12], were classified into three classes according to their CGs, the ORs increased as the CG advanced (ORs of [(HG III + HG IV) − CG I], [(HG III + HG IV) − CG II] and [(HG III + HG IV) − CG III] were 17.8, 14.2, and 130, respectively.). These results suggest that the combination of the CG and HG improved the accuracy in predicting the progression to ESRD, in comparison with either grade alone, in patients with IgAN. Furthermore, we defined four dialysis induction risk groups by grouping nine compartments into four groups based on the magnitude of ORs (Table 5). The logistic regression analysis revealed that the ORs for the risk of progression to ESRD significantly and progressively increased from the low-risk group to the super-high-risk group (Table 6). Of note, the patients in the super-high-risk group received various kinds of treatments, including corticosteroids (Table 7), suggesting that the extremely high OR of the super-high-risk group was not due to insufficient treatment.

Numerous studies have reported on the clinical and histological prognostic factors at the diagnosis of IgAN [2–6, 12, 15–19]. Most of these previous studies reported that severe proteinuria, a reduced renal function, and histological grading predicted disease progression, while some studies suggested that hypertension at RBx, severe hematuria, age, and gender were also prognostic factors. In our cohort, the multivariate logistic analysis revealed that proteinuria and the eGFR were significant independent variables, whereas hypertension, severe hematuria, age, and gender were not independently associated with progression to ESRD.

Prior to this analysis, several studies have used ROC analyses to investigate combinations of prognostic variables that could improve the accuracy in predicting future disease progression. In the Nord-Trondelag Health Study, a CKD classification that combined albuminuria and the eGFR improved prediction of ESRD [20]. Furthermore, in IgAN patients, the combination of proteinuria and the eGFR improved the accuracy in predicting the development of ESRD in comparison with either factor alone [14]. Thus, it is suggested that the inclusion of both proteinuria and the eGFR in the prediction model of the present analysis may help to improve the accuracy in predicting the risk of future ESRD in IgAN patients.

The optimal threshold values and classification may differ according to the patient background and the definition of the outcome. We selected our threshold values based on the following reasons. First, Imai et al. reported that Japanese IgAN patients are often diagnosed at a relatively early stage when they show asymptomatic proteinuria with microhematuria or isolated microhematuria, which can be found in the annual urinary screening system (kenshin), and many Japanese nephrologists believe that IgAN patients with early stage or mild proteinuria respond readily to treatment with RAS inhibitors or corticosteroids, while those with severe proteinuria (> 1.0 g/day) and a reduced creatinine clearance < 70 ml/min are often resistant to these treatments [21]. In addition, they also hypothesized that a therapeutic ‘golden period’ may exist when patients have moderate proteinuria < 1.0 g/day [21]. Furthermore, some IgAN patients with even mild proteinuria (< 0.4 g/day) or early stage IgAN showed a progressive course [22, 23]. Thus, we regarded sustained proteinuria at > 0.5 g/day as the level at which treatment should be initiated. Second, CKD is defined based on a GFR of 60 ml/min/1.73 m^2^, and various clinical events are associated with a GFR of < 60 ml/min/1.73 m^2^ for a period > 3 months—even in the absence of known structural alterations [24]. Third, the combination of the threshold values of UPE 0.5 g/day and eGFR 60 ml/min/1.73 m^2^ produced a suitable prognostic-predictive equation [threshold score (= -1.86) = 0.722 + 0.364 x UPE − 0.046 × eGFR] [14]. Thus, we selected UPE 0.5 g/day and eGFR 60 ml/min/1.73 m^2^ as the threshold values in the present analysis. Although these threshold values might not have been statistically optimal for the cohort of the present analysis, by dividing patients into three clinical grades using these values, the ORs for the risk of progression to ESRD were found to increase significantly from CG I to CG III (Table 4).

The *KDIGO 2012 Clinical Practice Guidelines*, which are accepted worldwide, reported the prognostic classification of CKD [24]. The classification consisted of 3 parameters (the cause of CKD, the category of GFR, and the category of albuminuria), and a GFR–albuminuria grid reflected the risk of CKD progression. Although the classification clearly showed that the risk of CKD progression increased with an advancement in the GFR and/or albuminuria categories, this classification system consisted of clinical parameters alone. In addition to the clinical prognostic parameters, various histological parameters are correlated with the renal prognosis in IgAN patients. However, the previous studies by us [12] and others [1] showed that nearly 10% of IgAN patients with the lowest histological grade or minor glomerular lesions progressed to ESRD. The discrepancies between the minor glomerular injury and progression to ESRD suggested that evaluating the prognosis of IgAN based on histological parameters alone is associated with some limitations. This led us to create better prognostic models that combined predictive clinical variables with the histological grades. Aside from our analysis, Barbour et al. recently reported that the risk prediction in IgAN could be significantly improved by adding the histological severity (Oxford MEST) to the clinical data (proteinuria) at RBx [25], suggesting the validity of our strategy.

The present analysis is associated with several limitations. First, the present analysis evaluated prognostic clinical and histological parameters at RBx and constructed the grading system. However, we could not fully clarify the effects of each therapy on the renal outcome because of the limited sample size and design of a retrospective case–control study. Second, because the clinical data were only available at the time of RBx, and at the end of the follow-up period, the assessments of the clinical course were limited. Thus, a further long-term prospective study with a large sample size will be necessary to assess the therapeutic effects on the renal outcome, the validity and reliability of the present grading system in IgAN.

In summary, the combination of the clinical grade and the histological grade improved the accuracy with which the risk of progression to ESRD could be predicted in IgAN patients in comparison with the clinical grade or the histological grade alone. The results suggest that our grading system for predicting the long-term prognosis of IgAN may be useful for the management of individual patients with IgAN.

## References

[1] Koyama A, Igarashi M, Kobayashi M (1997). Natural history and risk factors for immunoglobulin A nephropathy in Japan. Research Group on Progressive Renal Disease. Am J Kidney Dis.

[2] D’Amico G (2004). Natural history of idiopathic IgA nephropathy and factors predictive of disease outcome. Semin Nephrol.

[3] Roufosse CA, Cook HT (2009). Pathological predictors of prognosis in immunoglobulin A nephropathy: a review. Curr Opin Nephrol Hypretens.

[4] D’Amico G (2000). Natural history of idiopathic IgA nephropathy: role of clinical and histological prognostic factors. Am J Kidney Dis.

[5] Lv J, Shi S, Xu D (2013). Evaluation of the Oxford classification of IgA nephropathy: a systematic review and meta-analysis. Am J Kidney Dis.

[6] Cattran DC, Coppo R, Cook HT, Working Group of the International, IgA, Nephropathy Network and the Renal Pathology Society (2009). The Oxford classification of IgA nephropathy: rationale, clinicopathological correlations, and classification. Kidney Int.

[7] Haas M, Verhave JC, Liu ZH (2017). A multicenter study of the predictive value of crescents in IgA nephropathy. J Am Soc Nephrol.

[8] Yuzawa Y, Yamamoto R, Takahashi K (2016). Evidence-based clinical practice guidelines for IgA nephropathy 2014. Clin Exp Nephrol.

[9] Matsuo S, Kawamura T, Joh K (2011). Clinical guides for immunoglobulin A (IgA) nephropathy in Japan, third version. Jpn J Nephrol.

[10] Berthoux FC, Mohey H, Afiani A (2008). Natural history of primary IgA nephropathy. Semin Nephrol.

[11] Reich HN, Troyanov S, Scholey JW (2007). Toronto glomerulonephritis registry: remission of proteinuria improves prognosis in IgA nephropathy. J Am Soc Nephrol.

[12] Kawamura T, Joh K, Okonogi H (2013). A histologic classification of IgA nephropathy for predicting long-term prognosis: emphasis on end-stage renal disease. J Nephrol.

[13] Sato R, Joh K, Komatsuda A (2015). Validation of the Japanese histologic classification 2013 of immunoglobulin A nephropathy for prediction of long-term prognosis in a Japanese single-center cohort. Clin Exp Neprhol.

[14] Okonogi H, Utsunomiya Y, Miyazaki Y (2011). A predictive clinical grading system for immunoglobulin A nephropathy by combining proteinuria and estimated glomerular filtration rate. Nephron Clin Pract.

[15] Haas M (1997). Histologic subclassification of IgA nephropathy: a clinicopathologic study of 244 cases. Am J Kidney Dis.

[16] Manno C, Strippoli GFM, D’Altri C (2007). A novel simpler histological classification for renal survival in IgA nephropathy: a retrospective study. Am J Kidney Dis.

[17] Trimarchi H, Barratt J, Cattran DC (2017). Oxford Classification of IgA nephropathy 2016: an update from the IgA Nephropathy Classification Working Group. Kidney Int.

[18] Katafuchi R, Kiyoshi Y, Oh Y (1998). Glomerular score as a prognosticator in IgA nephropathy: its usefulness and limitation. Clin Nephrol.

[19] Lee HS, Lee MS, Lee SM (2005). Histological grading of IgA nephropathy predicting renal outcome: revisiting H.S. Lee’s glomerular grading system. Nephrol Dial Transplant.

[20] Hallan SI, Rits E, Lydersen S (2009). Combining GFR and albuminuria to classify CKD improves prediction of ESRD. J Am Soc Nephrol.

[21] Imai H, Miura N (2013). A treatment dilemma in adult immunoglobulin A nephropathy: what is the appropriate target, preservation of kidney function or induction of clinical remission?. Clin Exp Nephrol.

[22] Szeto CC, Lai FM, To KF (2001). The natural history of immunoglobulin A nephropathy among patients with hematuria and minimal proteinuria. Am J Med.

[23] Shen P, He L, Li Y (2007). Natural history and prognostic factors of IgA nephropathy presented with isolated microscopic hematuria in Chinese patients. Nephron Clin Pract.

[24] No authors listed (2013). Chapter 1: definition and classification of CKD. Kidney Int Suppl.

[25] Barbour SJ, Espino-Hemandez G, Reich HN (2016). The MEST score provides earlier risk prediction in IgA nephropathy. Kidney Int.

